# Improving the Rice Photosynthetic Efficiency and Yield by Editing *OsHXK1* via CRISPR/Cas9 System

**DOI:** 10.3390/ijms22179554

**Published:** 2021-09-02

**Authors:** Shaoyan Zheng, Chanjuan Ye, Jingqin Lu, Jiamin Liufu, Lin Lin, Zequn Dong, Jing Li, Chuxiong Zhuang

**Affiliations:** 1 State Key Laboratory for Conservation and Utilization of Subtropical Agro-Bioresources, College of Life Sciences, South China Agricultural University, Guangzhou 510642, China; zhengsy1229@163.com (S.Z.); chanjuanye@163.com (C.Y.); ljq110328@163.com (J.L.); liufujiamin@163.com (J.L.); 18133687842@163.com (L.L.); dzqaug15@163.com (Z.D.); lijing@scau.edu.cn (J.L.); 2 Guangdong Laboratory for Lingnan Modern Agriculture, South China Agricultural University, Guangzhou 510642, China

**Keywords:** *OsHXK1*, high yield, high photosynthetic efficiency, CRISPR/Cas9, photosynthesis-related gene expression

## Abstract

Rice (*Oryza sativa* L.) is an important food crop species in China. Cultivating high-yielding rice varieties that have a high photosynthetic efficiency is an important goal of rice breeding in China. In recent years, due to the continual innovation of molecular breeding methods, many excellent genes have been applied in rice breeding, which is highly important for increasing rice yields. In this paper, the hexokinase gene *OsHXK1* was knocked out via the CRISPR/Cas9 gene-editing method in the *indica* rice varieties Huanghuazhan, Meixiangzhan, and Wushansimiao, and *OsHXK1*-CRISPR/Cas9 lines were obtained. According to the results of a phenotypic analysis and agronomic trait statistics, the *OsHXK1*-CRISPR/Cas9 plants presented increased light saturation points, stomatal conductance, light tolerance, photosynthetic products, and rice yields. Moreover, transcriptome analysis showed that the expression of photosynthesis-related genes significantly increased. Taken together, our results revealed that knocking out *OsHXK1* via the CRISPR/Cas9 gene-editing method could effectively lead to the cultivation of high-photosynthetic efficiency and high-yielding rice varieties. They also revealed the important roles of *OsHXK1* in the regulation of rice yield and photosynthesis.

## 1. Introduction

Rice (*Oryza sativa* L.) is a major crop species that provides nearly a quarter of the dietary energy supply for people worldwide [1,2]. Increasing rice yields has always been a major goal of scientists. Because 90~95% of energy is produced by fixing carbon dioxide through photosynthesis, this process is the main factor determining rice yield [3]. Moreover, photosynthesis is considered to be the key way to improve the yield potential of major crop species [4]. However, thus far, photosynthesis has only slightly improved crop yields, and the levels are still far below the expected levels [5]. Previous research has shown that the factors limiting photosynthetic activity include light reactions, dark reactions, and metabolite transport from the source to sink tissue [6].

Photosynthesis is the process in which plants, algae, and some bacteria use light energy to reduce carbon dioxide and H_2_O to carbohydrates, releasing oxygen and producing ATP, NADPH, and H^+^ [7,8,9,10,11]. In addition to synthetic autotrophism, photosynthesis provides energy for all life on Earth [12]. To achieve the best efficiency of energy conversion, the light and dark reactions of photosynthesis need to be highly balanced [13,14]. As plants are fixed life forms, they are completely exposed to the surrounding environment. Any abiotic and biological factors that affect the daylighting or enzyme efficiency of the Calvin Benson cycle may lead to an imbalance between light and dark reactions, resulting in a change in photosynthetic efficiency. Therefore, photosynthesis is a sensitive system, and changes in the photosynthetic rate also reflect changes in the environment [15,16,17,18,19]. To achieve high rice yields, it is necessary to coordinate the source-sink-flow relation. Through simple pyramiding breeding, different excellent alleles have been aggregated with the goal of improving plant leaf morphology and significantly expanding storage capacity [20,21]. We should focus on improving the overall function of varieties and designing ideal physiological and ecological models to ensure a sufficient source-sink relation in new varieties to achieve high rice yields [22].

One of the most important ways to increase yield through photosynthesis is through the regulation of sugar metabolism or assimilate translocation [21,23,24]. Photosynthetic products have a negative feedback effect on photosynthesis. Therefore, increasing sugar synthesis in source organs has been indicated to be a strategy to increase plant yields. Enzymes directly or indirectly involved in the conversion of sugar to starch are often used to increase sink strength so that plants distribute more assimilates to seeds, tubers, and other organs [25,26]. A large number of studies have shown that hexokinase (HXK)-phosphorylated sugars in plants are also involved in the regulation of photosynthesis-related gene expression [27]. Therefore, HXK is considered an important sensor of leaf sugar. Recently, many studies have focused on trehalose-6-phosphate (T6P), which plays a central role in sugar signaling in plants, regulating sucrose distribution and metabolism [28,29,30,31]. Only when assimilates are directed to the required plant organs (such as rice grains) can the yield be increased by increasing photosynthesis [32,33].

HXK is a ubiquitous protein in all organisms. It plays an important role in metabolism, glucose signal transduction, and phosphorylation of glucose and fructose [34]. HXKs constitute a class of multifunctional proteins that play an important role in promoting germination, inhibiting seedling formation under high glucose concentrations, promoting vegetative growth and flowering, restoring fertility, and transducing senescence signals [32,35,36,37,38,39,40,41]. Therefore, by combining the biochemical characteristics, localization, and sensing ability of HXKs with other sugar-sensitive pathways, researchers can increase plant productivity [35]. HXKs can regulate photosynthetic activity by inhibiting the expression of several photosynthesis-related genes or by regulating enzymes that use carbon molecules to supply biosynthetic pathways and generate energy [42,43,44]. However, the function of HXKs affects not only photosynthetic tissues but also sink tissues [45]. Studies have shown that HXKs play an important role in different stages of plant development [35]. The mechanism by which HXK affects all stages of the plant life cycle may lie in the regulation of glucose abundance signals in several hormone signaling pathways, such as the auxin (AUX) [46], cytokinin (CK) [47], abscisic acid (ABA) [44], gibberellic acid (GA) [48], and brassinosteroid (BR) [49] pathways and even the growth regulator melatonin (MT) pathway [35]. Overexpression of *OsHXK5*, *OsHXK6,* or their noncatalytic mutant alleles in high-glucose media can restore the glucose-sensitive seedling development-arrested phenotype in Arabidopsis *gin2-1* plants. All transgenic *gin2-1* plants overexpressing *OsHXK5*, *OsHXK6,* or mutant alleles inhibited the expression of photosynthesis-related genes in high-glucose media. However, transgenic rice plants overexpressing *OsHXK5* or *OsHXK6* are hypersensitive, resulting in seedling growth retardation and inhibition of *RBC* genes upon treatment with glucose [50,51,52,53].

At present, the population is increasing, cultivated land is decreasing, and the environment is deteriorating. Rice production is facing severe challenges due to pollution and frequent extreme weather disasters. It is urgent to apply new technology to improve the problem of rice yield [54]. Genome editing is a newly developed technology that can modify crop genomes accurately [55]. Gene editing refers to the use of site-specific nucleases (SSNs), which can introduce specific double strand breaks (DBSs) into specific genomic regions or targets, resulting in the activation of the host DNA repair pathway. However, in the absence of homologous repair templates, target DNA is repaired through the nonhomologous end joining (NHEJ) pathway or homologous recombination (HR), which often leads to the production of dysfunctional alleles and targeted mutations [54]. The CRISPR/Cas9 system has become one of the most popular and powerful genome editing tools because of its simplicity, efficiency, and versatility [2,54]. The CRISPR/Cas9 system is an effective way to improve crop yield, stress resistance, nutrient utilization, insect resistance, and herbicide resistance [55,56,57].

In this study, we investigated the effects of *OsHXK1* on rice yield. We constructed an *OsHXK1* targeting vector via clustered, regularly interspaced, short palindromic repeat (CRISPR)/CRISPR-associated 9 (Cas9) technology and transformed the construct into several *indica* rice varieties, including Huanghuazhan (HHZ), Meixiangzhan (MXZ), and Wushansimiao (WSM). The results showed that knocking out *OsHXK1* by the CRISPR/Cas9 system significantly increased the rice yield and photosynthetic efficiency. Our study provides a theoretical basis for revealing the molecular mechanism through which *OsHXK1* regulates rice yield and photosynthesis, as well as the breeding of high-quality rice varieties.

## 2. Results

### 2.1. Mutation Characteristics of OsHXK1-CRISPR/Cas9 Plants

In previous studies, we found that *OsHXK1* overexpression can lead to decreased rice fertility [40]. To further analyze the effects of *OsHXK1* on rice yield, we used CRISPR/Cas9 gene editing technology to knock out *OsHXK1*. To analyze whether the excellent phenotypic characteristics caused by knocking out *OsHXK1* can be present in other rice varieties, we transformed the *OsHXK1* CRISPR/Cas9 vector into the main *indica* rice varieties used in China, including HHZ, MXZ, and WSM.

To analyze the mutations in *OsHXK1*-Cas9 lines of the three varieties, six homozygous mutant lines with successful gene editing were filtered through DNA extraction and Sanger sequencing (Figure S1). The transgenic lines obtained from HHZ were named CH1/HZ and CH5/HZ; the lines obtained from MXZ were named CH1/MZ and CH3/MZ; and the lines obtained from WSM were named CH2/W and CH3/W. The sequencing results showed that there were homozygous mutations in each variety. To test for possible off-target effects, we analyzed the genomes of *OsHXK1*-Cas9-transformed plants by the use of CRISPR-GE (http://skl.scau.edu.cn/offtarget/ accessed on 12 April 2020) and predicted possible off-target regions. We then performed PCR to amplify and isolate these sequences and applied Sanger sequencing and bioinformatics analysis to identify the off-target effects at these sites. No mutations were found in either *OsHXK1*-Cas9-transformed plant, suggesting that we avoided off-target effects by carefully selecting the target site (Figure S2).

### 2.2. OsHXK1-Cas9 Plants Show Significantly Improved Rice Yield-Associated Characteristics

**Figure 1:**
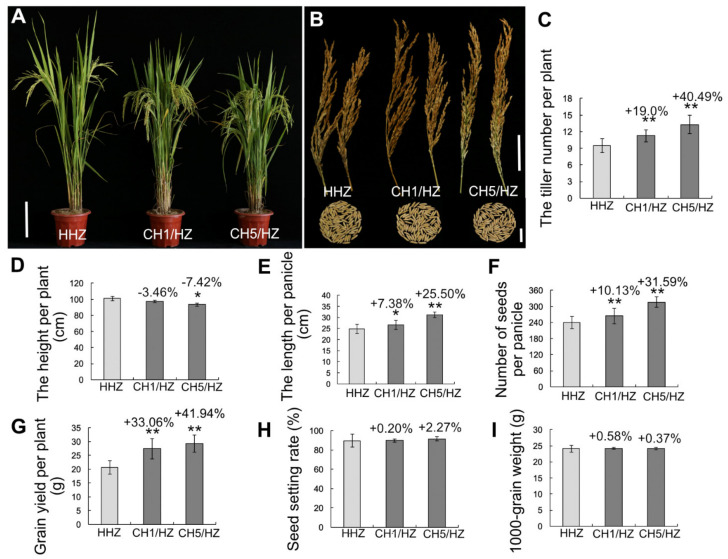
Yield traits of HHZ and *OsHXK1-Cas9* plants seeded in the fall. (**A**) Images of HHZ, CH1/HZ, and CH5/HZ plants at the flowering stage. Bar = 20 cm. (**B**) Comparisons of panicles and 100 grains of HHZ, CH1/HZ, and CH5/HZ plants. Bar = 5 cm (top), 2 cm (bottom). (**C**) Tiller number of HHZ, CH1/HZ, and CH5/HZ plants. (**D**) Height of HHZ, CH1/HZ, and CH5/HZ plants. (**E**) Length per panicle of HHZ, CH1/HZ, and CH5/HZ plants. (**F**) Number of grains per panicle of HHZ, CH1/HZ, and CH5/HZ plants. (**G**) Single-plant yield of HHZ, CH1/HZ, and CH5/HZ plants. (**H**) Seed setting rate of HHZ, CH1/HZ, and CH5/HZ plants. (**I**) 1000-grain weight of HHZ, CH1/HZ, and CH5/HZ plants. The data are the means ± SDs; *, 0.01 ≤ *p* < 0.05; **, 0.001 ≤ *p* < 0.01 according to Student’s *t*-test.

**Figure 2:**
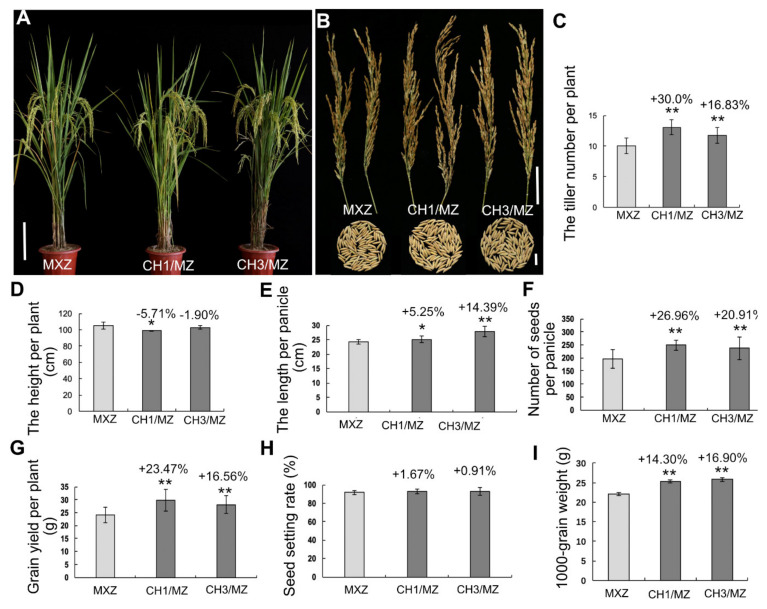
Yield traits of MXZ and *OsHXK1-Cas9* plants seeded in the fall. (**A**) Images of MXZ, CH1/MZ, and CH3/MZ plants at the flowering stage. Bar = 20 cm. (**B**) Comparisons of panicles and 100 grains of MXZ, CH1/MZ, and CH3/MZ plants. Bar = 5 cm (top), 2 cm (bottom). (**C**) Tiller number of MXZ, CH1/MZ, and CH3/MZ plants. (**D**) Height of MXZ, CH1/MZ, and CH3/MZ plants. (**E**) Length per panicle of MXZ, CH1/MZ, and CH3/MZ plants. (**F**) Number of grains per panicle of MXZ, CH1/MZ, and CH3/MZ plants. (**G**) Single-plant yield of MXZ, CH1/MZ, and CH3/MZ plants. (**H**) Seed setting rate of MXZ, CH1/MZ, and CH3/MZ plants. (**I**) 1000-grain weight of MXZ, CH1/MZ, and CH3/MZ plants. The data are the means ± SDs; *, 0.01 ≤ *p* < 0.05; **, 0.001 ≤ *p* < 0.01 according to Student’s *t*-test.

**Figure 3:**
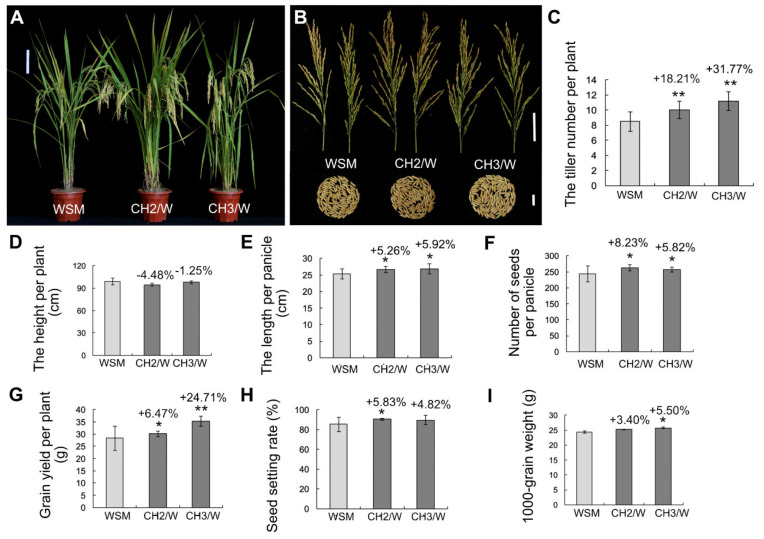
Yield traits of WSM and *OsHXK1-Cas9* plants seeded in the fall seedings. (**A**) Images of WSM, CH2/W, and CH3/W plants at the flowering stage. Bar = 20 cm. (**B**) Comparisons of panicles and 100 grains of WSM, CH2/W, and CH3/W plants. Bar = 5 cm (top), 2 cm (bottom). (**C**) Tiller number of WSM, CH2/W, and CH3/W plants. (**D**) Height of WSM, CH2/W, and CH3/W plants. (**E**) Length per panicle of WSM, CH2/W, and CH3/W plants. (**F**) Number of grains per panicle of WSM, CH2/W, and CH3/W plants. (**G**) Single-plant yield of WSM, CH2/W, and CH3/W plants. (**H**) Seed setting rate of WSM, CH2/W, and CH3/W plants. (**I**) 1000-grain weight of WSM, CH2/W, and CH3/W plants. The data are the means ± SDs; *, 0.01 ≤ *p* < 0.05; **, 0.001 ≤ *p* < 0.01 according to Student’s *t*-test.

We grew *OsHXK1*-CRISPR/Cas9 plants in the field. To determine the effects of *OsHXK1* on yield components, we measured the agronomic characteristics of *OsHXK1*-Cas9 lines and WT plants sown during spring and fall seeding. The results showed that, compared with HHZ/MXZ/WSM, the *OsHXK1*-Cas9 lines CH1/HZ and CH5/HZ, CH1/MZ and CH3/MZ, CH2/W and CH3/W grew better and had more tillers (Figure 1A,B, Figure 2A,B and Figure 3A,B). During fall seeding, compared with that for the WT, the number of effective tillers for the *OsHXK1*-Cas9 lines CH1/HZ and CH5/HZ increased by 19.00% and 40.49%, respectively, those for CH1/MZ and CH3/MZ increased by 16.83% and 30.00%, respectively, and those for CH2/W and CH3/W increased by 18.21% and 31.77%, respectively (Figure 1C, Figure 2C and Figure 3C). Compared to the plant height of HHZ, those of CH1/HZ and CH5/HZ decreased by 3.46% and 7.42%, respectively, but the average plant height of CH1/MZ and CH3/MZ decreased slightly compared with that of MXZ, and the height of CH2/W and CH3/W decreased by 4.48% and 1.25%, respectively, compared with that of WSM (Figure 1D, Figure 2D and Figure 3D). The panicle lengths of CH1/HZ and CH5/HZ increased by 7.38–25.50% (Figure 1E), the average main panicle length of MXZ was approximately 24.33 cm, and those of CH1/MZ and CH3/MZ were 25.16 cm and 27.83 cm, respectively, equal to increases of 5.25% and 14.39%, respectively (Figure 2E). The CH2/W and CH3/W increased by 5.26% and 5.92%, respectively (Figure 3E). The number of grains per panicle of CH1/HZ and CH5/HZ increased by 10.13–31.59% (Figure 1F), and those of CH1/MZ and CH3/MZ were 249 and 237, equal to increases of 26.96% and 20.91%, respectively, compared to that of MXZ (approximately 196) (Figure 2F). Similarly, the number of grains per panicle of CH2/W and CH3/W increased slightly (by 8.23% and 5.82%, respectively) compared to that of WSM (Figure 3F).

According to the single-plant yield statistics, the results showed that the grain yield per plant of CH1/HZ and CH5/HZ increased by 33.06% and 41.94%, respectively (Figure 1G), and CH1/MZ and CH3/MZ increased by 16.56–23.47% (Figure 2G). Compared with that of WSM, the grain yield of CH2/W and CH3/W increased by 6.47% and 24.71%, respectively (Figure 3G). However, the 1000-grain weight and seed setting rate increased by approximately 0.37–16.90% and 0.20–5.83%, respectively (Figure 1H,I, Figure 2H,I and Figure 3H,I). During spring seeding, the tiller number, panicle length, and panicle number also obviously increased (Figures S3–S5). To determine whether the function of *OsHXK1* can improve the quality of rice in addition to yield, the *OsHXK1*-Cas9 lines of the three varieties and WT rice were sent to the China Rice Research Institute for determination and identification, and the results of the identification report showed that the rice quality of the three varieties did not change (Tables S1–S3). These results indicated that the agronomic traits of the three varieties could be significantly optimized by knocking out *OsHXK1* and that the rice quality was not inferior to that of the WT.

### 2.3. OsHXK1-Cas9 Plants Exhibit Increased Photosynthetic Efficiency

**Figure 4:**
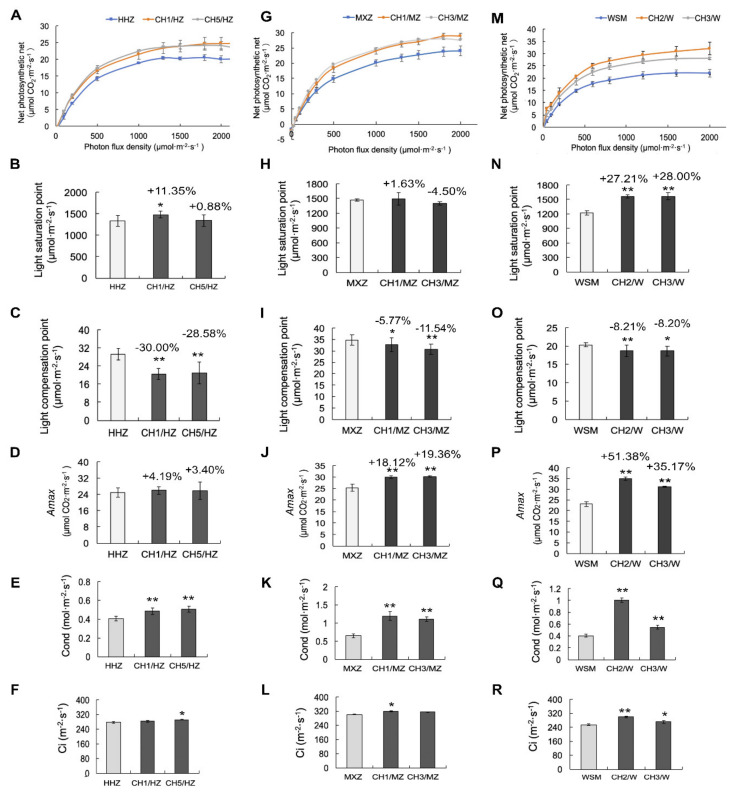
Analysis of photosynthesis properties of HHZ, MXZ, WSM, and *OsHXK1-Cas9* plants. (**A**,**G**,**M**), Light-response curves were generated for HHZ, MXZ, WSM, CH1/HZ, and *OsHXK1-Cas9* plants under normal air conditions. (**B**–**D**,**H**–**J**,**M**–**P**), Statistics of the LSP, LCP, and *A*_max_ of HHZ, MXZ, WSM, and CH1/HZ, and *OsHXK1-Cas9* plants. All measurements were performed using the flag leaves of rice at the flowering stage. Shown are the means ± SDs; *n* = 3; *, 0.01 ≤ *p* < 0.05; **, 0.001 ≤ *p* < 0.01, according to Student’s *t*-test. (**E**,**F**,**K**,**L**,**Q**,**R**), Cond and Ci of HHZ, MXZ, WSM, and *OsHXK1-Cas9* plants. Shown are the means ± SDs (*n* = 3); *, 0.01 ≤ *p* < 0.05; **, 0.001 ≤ *p* < 0.01, according to Student’s *t*-test.

Photosynthesis is the basis of crop yield. We knocked out *OsHXK1* in three varieties to improve rice yield (Figure 1, Figure 2 and Figure 3). To analyze the relationship between yield and photosynthesis in rice, we generated light response curves for the WT and *OsHXK1*-Cas9 plants of the three varieties. The *OsHXK1*-Cas9 plants of the three varieties presented a higher Pn according to the light response curves (Figure 4A,G,M). Compared to the HHZ plants, the CH1/HZ and CH5/HZ plants presented a higher LSP and *A*_max_, which increased by 0.88–11.35% and 3.40–4.19%, respectively (Figure 4B,D), and the LCP decreased by 28.58–30.00% (Figure 4C).

The LSP and *A*_max_ increased by −4.50–1.63% and 18.12–19.36% in CH1/MZ and CH3/MZ (Figure 4H,J), respectively, and the LCP decreased by 5.77–11.54% (Figure 4I) compared with those of MXZ. Compared with the WSM plants, the *OsHXK1*-Cas9 plants presented LSP and *A*_max_ increases of 27.21–28.00% (Figure 4N) and 35.17–51.38%, respectively (Figure 4P). The LCP decreased by approximately 8.20% (Figure 4O), and the intercellular carbon dioxide concentration (Ci) and stomatal conductance (Cond) of the *OsHXK1*-Cas9 plants also obviously increased (Figure 4E,F,K,L,Q,R). We also observed the stomatal apertures at midday by the scanning electron microscopy (SEM), and most stomata of the wild type were closed (Figure S6A,D,G,J,M,P), while some of the *OsHXK1*-Cas9 plants were not completely closed (Figure S6B,C,E,F,H,I,K,L,N,O,Q,R). Taken together, these results suggested that the *OsHXK1*-Cas9 plants could regulate the stomatal apertures on stomatal conductance to significantly improve photosynthesis and obviously accumulated increased amounts of photosynthetic products in the HHZ and MXZ varieties.

### 2.4. OsHXK1-Cas9 Plants Exhibit an Increased Abundance of Photosynthetic Products

**Figure 5:**
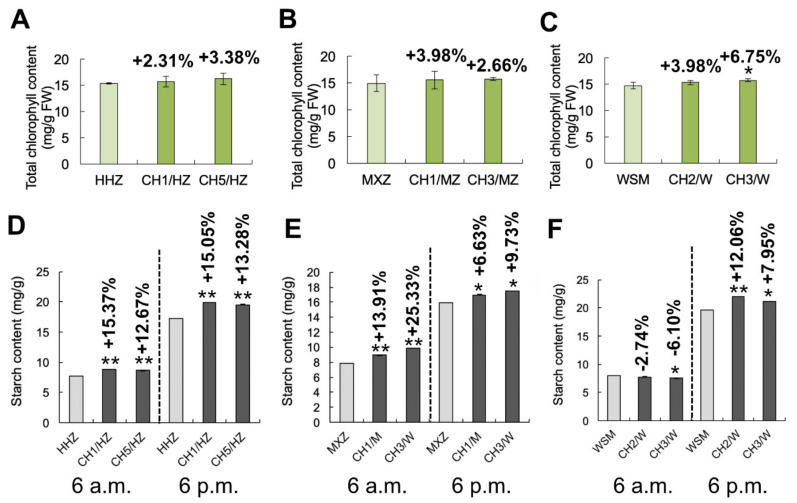
Measurement of chlorophyll and total starch contents in the three varieties. (**A**–**C**) The total chlorophyll contents in HHZ, MXZ, WSM, CH1/HZ, and *OsHXK1-Cas9* plants during the flowering stage. Shown are the means ± SDs; *, *p* < 0.05; **, *p*< 0.01 according to Student’s *t*-test. (**D**–**F**) Total starch contents in leaves of HHZ, MXZ, WSM, and CH1/HZ and *OsHXK1-Cas9* plants at the flowering stage in the morning and evening. Shown are the means ± SDs; *, 0.01 ≤ *p* < 0.05; **, 0.001 ≤ *p* < 0.01, according to Student’s *t*-test.

Previous studies have shown that the chlorophyll content in plant leaves is closely related to photosynthesis, and the change in chlorophyll content can also reflect the level of plant photosynthetic efficiency. To determine the relationship between *OsHXK1* and the improvement in photosynthesis, we analyzed the chlorophyll contents of WT and *OsHXK1-Cas9* leaves. Compared with that in the WT, the leaf chlorophyll content in the *OsHXK1-Cas9* lines of all varieties increased (Figure 5A–C). Plants can synthesize starch via photosynthesis during the day and store it in their leaves [23]. At night, a large amount of starch can be broken down by respiration. Therefore, the accumulation of photosynthetic products during the day and consumption by respiration at night can indicate the strength of photosynthesis. We compared the total starch content between WT and *OsHXK1-Cas9* leaves at 6 a.m. and 6 p.m. and found that in HHZ and MXZ the total starch content of *OsHXK1-Cas9* leaves increased significantly at 6 p.m. compared with that at 6 a.m. These results indicated that the products of photosynthesis in the daytime increased in these two varieties, and the amount of products consumed by respiration at night was lower (Figure 5D–F). Taken together, these results indicated that the *OsHXK1-Cas9* plants presented significantly improved photosynthesis, accumulating enough photosynthetic products and energy for plant growth and development and ultimately increasing rice yield.

### 2.5. OsHXK1 Promoted the Expression of Genes Related to Photosynthesis

**Figure 6:**
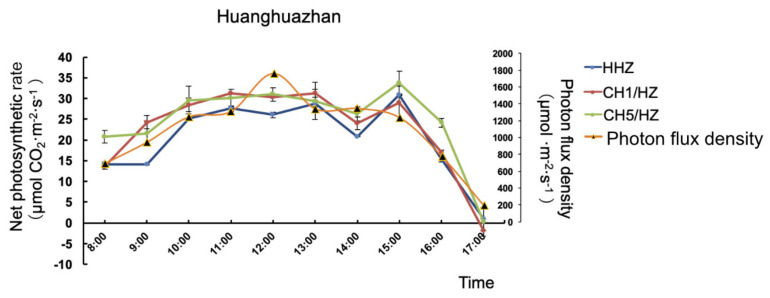
Diurnal curves of the Pn of HHZ, CH1/HZ, and CH5/HZ plants. Diurnal curves of the Pn of the HHZ, CH1/HZ, and CH5/HZ plants in June in Guangzhou, China (temperature range of 28~36 °C under normal air conditions (CO_2_ concentration of approximately 400 mmol·mol^−1^, relative humidity of 50~75%)), were generated. Data shown are the means ± SDs.

By measuring the dynamic diurnal photosynthetic curve of HHZ, we found that the WT (HHZ) exhibited a midday depression phenomenon. Compared with that of the WT plants, the photosynthetic efficiency of the *OsHXK1-Cas9* plants (CH1/HZ and CH5/HZ) was still higher than that of the WT at 12 a.m. (Figure 6). Therefore, we selected leaves at 12 a.m. as samples for the preparation of RNA-seq samples to analyze the regulation of photosynthesis by *OsHXK1*.

**Figure 7:**
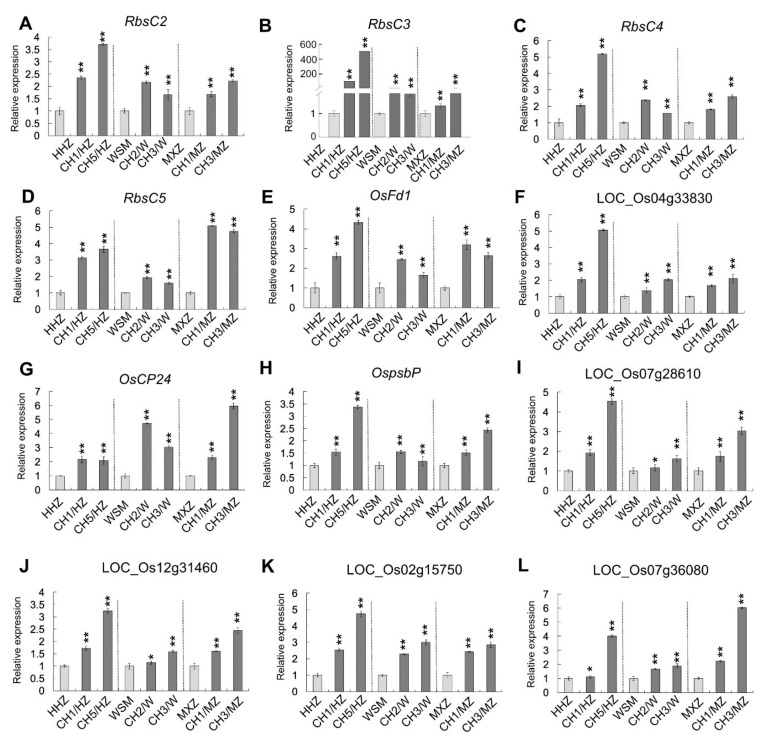
Expression analysis of photosynthesis-related genes in three varieties. (**A**–**L**) qRT-PCR analysis of the expression levels of photosynthesis-related genes in HHZ, MXZ, WSM, and *OsHXK1-Cas9* plants according to the RNA-seq data. *OsActin1*, *UQ5, GAPDH* were used as the internal reference genes. The data shown are the means ± SDs; *, 0.01 ≤ *p* < 0.05; **, 0.001 ≤ *p* < 0.01, according to Student’s *t*-test.

To determine the regulatory role of *OsHXK1* in terms of yield and photosynthesis, we selected the flag leaves of HHZ, CH1/HZ, and CH5/HZ plants at 12 a.m. to prepare the RNA-seq samples, analyze the transcriptome data to screen the DEGs between HHZ, CH1/HZ and CH5/HZ plants, and then analyze the regulatory network of *OsHXK1* in terms of its effects on yield and photosynthesis. By analyzing the transcriptome data, we found that the expression values of different genes in each comparison group (HHZ12-CH1/HZ12 and HHZ12-CH5/HZ12) clustered together (Figure S7). By screening the DEGs, we found that in the HHZ12-CH1/HZ12 group, 1072 genes were upregulated 0.6-fold and that 1338 genes were downregulated 0.6-fold (Figure S8A). Moreover, in the HHZ12-CH5/HZ12 group, 1845 genes were upregulated (log_2_(fold change (FC)) ≥ 0.6), and 2733 genes were downregulated (log_2_(FC) ≤ 0.6) (Figure S8B). A total of 703 genes were upregulated in both groups (log_2_(FC) ≥ 0.6) (Figure S8C). The DEGs were involved mainly in photosynthesis-related pathways, including carbon metabolism and fixation pathways, glycolysis, and the tricarboxylic acid (TCA) cycle (Figure S8D,E). The significantly expressed genes identified in the screening analysis of HHZ12-CH1/HZ12 and HHZ12-CH5/HZ12 were further analyzed, and the results showed that 34 genes were involved mainly in the regulation of the photosynthesis pathway (Figure S9, Table S4). By using qRT-PCR, we then verified the expression of several genes related to photosynthesis, photosystems and chlorophyll synthesis in HHZ, MXZ, and WSM. The results showed that, compared with those in the WT plants (HHZ, MXZ, WSM), all the identified genes involved in photosynthesis pathways in the *OsHXK1-Cas9* plants (CH1/HZ, CH5/HZ, CH1/MZ, CH3/MZ, CH2/W, CH3/W) were upregulated (Figure 7), indicating that *OsHXK1* may play an important role in the regulatory network of photosynthesis.

### 2.6. Haplotype Analysis of OsHXK1 in 930 Rice Varieties

In recent years, molecular biology has played an increasingly important role in the study of rice yield traits. Discovering and mining excellent alleles are important to increase rice yields and apply them to breeding practices. The results of this study showed that knocking out *OsHXK1* in three indica rice varieties could significantly increase rice yields, indicating that *OsHXK1* may have important value in breeding high-yielding rice. Therefore, this study aimed to identify the key single-nucleotide polymorphism (SNP) sites within *OsHXK1* through haplotype analysis, which will provide a theoretical basis for the subsequent mining of excellent alleles of *OsHXK1* and their application in production. We analyzed the *OsHXK1* coding DNA sequences (CDSs) of 930 rice varieties from 11 provinces via bioinformatics and found eight SNPs that could be classified as one of four haplotype variations, among which five SNPs altered the amino acid sequence (Figure S10A). These four haplotypes are helpful for improving the adaptability of rice in different regions.

We classified the 930 rice varieties from 11 provinces in China and analyzed their haplotypes for the *OsHXK1* gene. Haplotype analysis of polymorphisms in *OsHXK1* showed that the proportion of group I haplotypes was relatively high. There were 705 group I haplotypes in the 930 cultivars analyzed. Additionally, there were 181 group II haplotypes, 37 group III haplotypes, and 7 group IV haplotypes (Table S5). However, the haplotype distribution of *OsHXK1* was different among the varieties at different provinces (Figure S10B, Table S5). In Guangdong province, the proportions of group I haplotypes were relatively low, accounting for 34.3%, other provinces have more than 60% (Figure S10B).

There were three haplotypes of group I, group II, and group III among the 99 varieties in Guangdong Province, 42 varieties in Guangxi Province, 59 varieties in Zhejiang Province, 48 varieties in Hubei Province, and 31 varieties in Chongqing Province. Among the 82 varieties in Fujian Province, 48 in Jiangxi Province, and 318 in Hunan Province, there were four haplotypes: group I, group II, group III, and group IV. There were only two haplotypes (group I and group II) in 30 cultivars from Jiangsu Province and 29 cultivars from Anhui Province (Table S5).

## 3. Discussion

### 3.1. Knockout of OsHXK1 Can Improve Rice Photosynthesis

Increasing global temperatures and the frequent occurrence of extreme weather events pose great challenges to crop yields. Therefore, the cultivation of high-yielding and high-light-efficiency rice varieties is highly important for promoting the sustainable production and steady development of rice [58]. Rice is an important C3 plant species, and the development of rice varieties with high photosynthetic efficiency is a current research interest [59]. In the past, photosynthesis of C3 plants was improved mainly by improving photosynthetic carbon assimilation [60]. For example, the key enzyme-encoding genes of the photosynthetic pathway of C4 plant species were transferred into C3 plants for improvement [61]. Because C4 plants have strong heat resistance and drought resistance and have a set of CO_2_ concentration mechanisms, they can maintain high CO_2_ concentrations in their chloroplasts and can ensure a sufficient photosynthesis rate under adverse conditions [62,63]. The CO_2_ concentration mechanism in C3 crop species involves making full use of the CO_2_ released by photorespiration, improving the CO_2_ concentration in chloroplasts, and improving the photosynthetic efficiency of those crop species. This mechanism can also simulate the improvement of the heat resistance and drought resistance of C3 crop species, resulting in favorable results with no penalty [6]. Shen et al. (2019) generated a new chloroplastic photorespiratory bypass method in rice chloroplasts to alter the contents of glycolic acid, glyoxylic acid, oxalic acid, and carbon dioxide; reduce respiration; and improve photosynthetic efficiency [64]. However, the transport of assimilates from source to sink has always not improved in rice. The problem in which a high photosynthetic efficiency is not reflected in high rice yields has yet to be solved.

HXKs play central roles in the regulation of plant sugar metabolism and have been identified as sugar sensors involved in the plant glycolysis pathway [46,48,52]. Previous studies have shown that HXKs can mediate stomatal closure to affect photosynthesis via sugar induction and the ABA pathway [44]. These findings indicate a simple feedback inhibition mechanism in the response of stomata to sucrose. The sucrose concentration in the apoplast of guard cells might affect the relationship between the photosynthesis rate and the capacity to export photosynthetic products from leaves through the phloem [65,66]. In our study, the photosynthetic efficiency of the *OsHXK1-Cas9* lines in the HHZ, MXZ and WSM treatments increased, which is likely because the LSP and maximum net photosynthetic efficiency of the *OsHXK1-Cas9* lines of the three varieties significantly improved and because the Ci and Cond increased (Figure 4B–F,H–L,N–R). Moreover, with the increase in Ci and Cond of the *OsHXK1-Cas9* lines, those plants could fix more carbon dioxide and consume more water; moreover, photosynthesis increased under high light and resisted photoinhibition more effectively (Figure 5).

According to previous studies, overexpression of *AtHXK1* can inhibit plant growth and the expression of photosynthesis-related genes and can reduce the chlorophyll content, thus decreasing photosynthetic efficiency [25,46,67]. Compared with WT plants, plants in which *OsHXK1* was knocked out presented higher chlorophyll levels, a greater accumulation of photosynthetic products during the daytime, and a lower consumption of photosynthates via respiration at night (Figure 5). From the transcriptome data, the expression of genes related to photosynthesis, photosystems, and chlorophyll synthesis in the three varieties was upregulated (Figure 7); thus, the photosynthetic capacity of the *OsHXK1-Cas9* plants could adjust to the changing light environment, improving their photosynthetic efficiency. It has previously been shown that starch is synthesized in the leaves during the day from carbon fixed by photosynthesis and is consumed at night to support continued respiration, carbohydrate transport, and growth of plants [68]. Our results showed that, compared with the WT plants, the *OsHXK1-Cas9* plants exhibited a higher accumulation of photosynthetic products during the daytime and a lower consumption of products by respiration at night. Taken together, these results suggest that *OsHXK1* plays an important regulatory role in rice photosynthesis.

### 3.2. Knockout of OsHXK1 Obviously Increased Rice Yield Traits

As rice is an important food for more than half of the global population, the improvement of rice yield is still an important solution to the problem of food shortages caused by the rapid increase in the global population [1,69]. Hybrid rice breeding has long been the main way to increase rice yields. Mutations produced by natural variation provide good materials for the functional breeding of rice genes, but spontaneous mutations are random and cannot be predicted [5]. Although physical and chemical mutagenesis can improve mutation efficiency, mutation sites cannot be predicted, and there are many uncontrollable factors. CRISPR/Cas9 gene editing technology makes the acquisition of mutants more rapidly, that is, quicker and more directional, and can effectively solve the quagmire between gene functional research and breeding production needs [57,70,71,72].

The main factors affecting rice yield are total grain number per panicle, effective panicle number (degree of rice tillering), 1000-grain weight, and the seed setting rate, all of which are typical quantitative characteristics [73]. Rice tillering is an agronomic trait that determines the panicle number, which then affects rice grain yield [74]. *MONOCULM 1* (*MOC1*) controls rice tillering and functions in axillary meristem (AM) initiation and outgrowth during the vegetative and reproductive stages of rice [75,76,77]. Grain number is also an important characteristic that determines crop yield; grain number per panicle is an important factor determining rice yield and is related to grain weight per plant and panicle number. *Ghd7* belongs to the CCT gene family and was isolated by Xue et al. (2008); *Ghd7* is a major gene controlling plant height, heading date and grain number per panicle. Compared with that of WT plants, the height of *Ghd7* transgenic plants increased, and the number of spikelets per panicle increased significantly [78]. In addition, several genes, such as *GS3*, *GW2*, *GW5*, *DEP1*, *Gn1a*, and *TGW6*, affect yield mainly by regulating grain weight and size [79,80,81,82].

Our results showed that the yield of the *OsHXK1-*Cas9 plants of the three varieties (HHZ, MXZ, WSM) significantly increased, but the rice quality did not change (Figure 1, Figure 2 and Figure 3, Tables S1–S3). According to the statistical data of the agronomic traits of the WT and target mutant lines grown in plot tests, compared with the WT plants of the three varieties, the *OsHXK1-Cas9* lines (CH1/HZ, CH5/HZ, CH1/MZ, CH3/MZ, CH2/W, CH3/W) presented significantly greater total grains per panicle, effective tiller numbers panicle length, yield per plant and plot yield (Figure 1,  Figure 2 and Figure 3). The quality of starch in rice contributes greatly to food security, and the amylose content is closely related to the eating quality of rice. The amylose contents of the *OsHXK1-Cas9* lines were similar to those of the WT and were similar to those of rice with other degrees of quality (Tables S1–S3). Haplotype analysis of *OsHXK1* in 930 rice varieties from 11 provinces of China revealed that 5 SNPs altered the amino acid sequence (Figure S10 and Table S5). No haplotypes with base deletions were found in these varieties, indicating that *OsHXK1* is conserved in the various varieties, and the application of CRISPR/Cas9 gene-editing technology is more convenient for producing new haplotypes conducive to improving yield, which is highly important for accelerating rice production. Taken together, our results show that *OsHXK1* is a negative regulatory gene that affects the total number of grains per panicle and effective tiller number in rice.

## 4. Materials and Methods

### 4.1. Plant Materials and Growth Conditions

Wild-type (WT) rice plants (*Oryza sativa* L. ssp. *indica*), including HHZ, MXZ, WSM, and *OsHXK1*-CRISPR/Cas9 transgenic lines, were grown in paddy fields at South China Agricultural University, Guangzhou, China. The *OsHXK1*-CRISPR/Cas9 transgenic lines CH1/HZ, CH5/HZ, CH1/MZ, CH3/MZ, CH2/W, and CH3/W were identified via PCR and Sanger sequencing.

### 4.2. Characterization of Transgenic Plant Phenotypes

All the *indica* rice materials used in this experiment were planted in the field at South China Agricultural University, Guangzhou, China, during spring and fall seeding. At the mature stage, 15 plants of each material were randomly selected for statistical analysis of the main agronomic traits, including the total number of grains per panicle, panicle length per plant, plant height, effective tillers, 1000-grain weight, grain yield per plant, seed setting rate, and actual yield per plot (*n* = 50 plants). Multiple repeated mean values of each index were statistically analyzed. Each experiment was performed for at least three replications. The data are presented as the means ± SDs. The data analysis was performed via Student’s *t*-test. The results were considered statistically significant when *p* < 0.05 (*) and extremely significant when *p* < 0.01 (**). The scanning electron microscopy (SEM) images of stomatal apertures in *OsHXK1-Cas9* plants used dried leaf samples were mounted on an aluminum stub, coated with a thin layer of gold, and observed with a scanning electron microscope (SEM) (Carl Zeiss, EVO MA 15, Thornwood, NY). For phenotypic characterization, the plants were imaged with a digital camera (Canon 750D, Japan).

### 4.3. Vector Construction and Plant Transformation

*OsHXK1* target sequences were designed to generate an *OsHXK1-*CRISPR/Cas9 vector by using the pYLgRNA—*OsHXK1-*OsU3 sequence as described previously [40,57]. All the constructs were confirmed by sequencing and then introduced into *Agrobacterium tumefaciens* EHA105 cells. The constructs were then transformed into HHZ, MXZ, and WSM cells by the *Agrobacterium*-mediated transformation method [83]. All the primers used for vector construction are listed in Table S6.

### 4.4. RNA Extraction and qRT-PCR Analysis

Total RNA was extracted from various plant tissues using TRIzol reagent (Genstar, Beijing, China). Reverse transcription was then performed on 500 ng of total RNA using qPCR-RT Master Mix with gDNA Remover (Vazyme, Nanjing, China). Quantitative RT-PCR analysis was performed in conjunction with RealStar Green Fast Mixture (GenStar, Beijing, China) with a qTOWER3G Real-Time PCR Detection System (AnalytikJena, Germany), and the data were analyzed with the 2^−∆∆CT^ method [84]. The *OsActin1, UQ5, GAPDH* were used as internal reference to normalize the gene expression data [85]. All the data analyses were repeated at least three times. In the figures, asterisks were used to represent significant differences at *p* < 0.001 (***), *p* < 0.01 (**), and *p* < 0.05 (*). All specific primers for the qRT-PCR were designed using Primer 3 (http://primer3.ut.ee/ accessed on 25 July 2021) and NCBI Primer-blast (www.ncbi.nlm.nih.gov/tools/primer-blast/ accessed on 25 July 2021). A standard curve was created using a 5-fold dilution of all cDNAs, and amplification efficiency values ranged from 90.2% to 100.5% with typical correlation coefficients (R^2^ ranged from 0.981 to 0.999) were obtained for all primers. The PCR primers used for gene amplification are listed in Table S6.

### 4.5. RNA Sequencing (RNA-Seq) Analysis

The RNA samples used for transcriptome analysis in this research were prepared from fully expanded flag leaves at the flowering stage from WT (HHZ), CH1/HZ, and CH5/HZ plants grown under natural field conditions at 12 a.m. RNA-seq was performed by Beijing Genomics Institution (BGI; Shenzhen, China). Following cluster generation and library preparation, reads were generated on the BGISEQ-500 platform (BGI-Shenzhen, China). Differential gene expression between WT (HHZ) RNA-seq data and both CH1/HZ and CH5/HZ RNA-seq data collected at 12 a.m. was analyzed using the Dr. Tom platform (http://report.bgi.com accessed on 25 July 2021, BGI). *p* < 0.001 was considered significant. The differentially expressed genes (DEGs) in CH1/HZ and CH5/HZ versus HHZ are listed in Table S4.

### 4.6. Photosynthesis Analysis

Photosynthesis measurements were performed using an LI-6400-XT (LI-COR, USA) instrument and software according to a protocol described previously [64]. Fully expanded flag leaves at the flowering stage from plants grown in pots were used for determination. Diurnal curves of net photosynthetic rates (Pns) were generated under natural air and light conditions in October. For generation of the light response curves, the conditions were as follows: a leaf temperature of 30-36 °C; a relative humidity of 60%; a CO_2_ concentration of 400 mmol·mol^−1^; and a gradually increasing photosynthetic photon flux density (PPFD) from 0 to 2000 mmol·m^−2^·s^−1^ (controlled by an LI-COR-6400XT LED irradiation source). The light saturation point (LSP) and light-saturated photosynthetic rate (*A*_max_) calculated from the light response curves were fit by the “photosynthesis app” supplied by the LI-COR manufacturer. All the data analyses were repeated at least three times; the data are presented as the means ± SDs. The data analysis was performed via Student’s *t*-test. In the figures, asterisks were used to represent significant differences at *p* < 0.001 (***), *p* < 0.01 (**), and *p* < 0.05 (*).

### 4.7. Determination of Chlorophyll and Starch Contents

Fresh rice leaves (0.05 g) were collected, and the leaves were cut into 1 mm^2^ pieces with scissors (avoiding the middle vein), which were then put into a centrifuge tube containing 95% ethanol. After incubation in darkness, the leaves were shaken overnight and until their green color had completely diminished. The ethanol solution containing the chlorophyll was subsequently transferred to a 15 mL centrifuge tube. The residual chlorophyll in the original centrifuge tube was eluted completely with an ethanol solution. The chlorophyll solution in the 15 mL centrifuge tube was then reduced to 10 mL and mixed thoroughly. A standard enzyme instrument was used for detection, and three parallel experiments were established for the same experiment. The absorbance values were measured at 649 nm and 665 nm, and the chlorophyll concentrations were calculated. The formulas used are as follows:

C_a_ (chlorophyll a) = (13.95A_665_ − 6.8A_649_) ∗ V/W

C_b_ (chlorophyll b) = (24.96A_649_ − 7.32A_665_) ∗ V/W (1)

C_Total_ (total chlorophyll) = C_a_ + C_b_ = (18.16A_649_ + 6.63A_665_) ∗ V/W (2)

The starch content detection method used in this study was performed according to the instructions of the Solarbio starch content detection kit (#BC0705, Solarbio, Beijing). All experiments were repeated at least three times, and Student’s *t*-test was applied for statistical analysis. In the figures, asterisks were used to represent significant differences at *p* < 0.001 (***), *p* < 0.01 (**), and *p* < 0.05 (*).

## 5. Conclusions

In this study, we used the CRISPR/Cas9 gene-editing method to knock out *OsHXK1* in three *indica* varieties, HHZ, MXZ, and WSM, and obtained *OsHXK1*-*Cas9* plants. Our results showed that, compared with the WT plants, the *OsHXK1*-*Cas9* lines presented increased chlorophyll contents; increased expression of photosynthesis-related genes; and increased LSP, *A*_max_, and Cond. Moreover, the *OsHXK1*-*Cas9* lines presented increased photosynthetic efficiency, reduced consumption of photosynthetic products, and increased accumulation of photosynthetic products during the day. Statistical analysis of agronomic traits showed that knocking out *OsHXK1* mainly increased the grain number per panicle and single-plant yield, increasing rice yields. Moreover, transcriptome sequencing analysis showed that *OsHXK1* upregulated the expression mainly of photosynthesis-related genes, thereby driving improvements in photosynthesis. These results suggest that *OsHXK1* plays an important role in the regulation of photosynthesis to increase rice yields.

## Data Availability

All data are provided as figures, tables, and supplementary information, which are included in this paper. The sequence data used in this study can be found in the Rice Annotation Project (https://rapdb.dna.affrc.go.jp/viewer/gbrowse accessed on 25 July 2021) and have been deposited in the GenBank database.

## Supplementary Materials

The following are available online at https://www.mdpi.com/article/10.3390/ijms22179554/s1.

## Abbreviations

CRISPR: clustered regularly interspaced short palindromic repeats; Cas9: CRISPR-associated protein 9; DEGs: differentially expressed genes; KEGG: Kyoto Encyclopedia of Genes and Genomes; GO: gene ontology.
