# Supplementary material for: Improving the Rice Photosynthetic Efficiency and Yield by Editing OsHXK1 via CRISPR/Cas9 System
Source: Int J Mol Sci. 2021 Sep 2;22(17):9554. doi: 10.3390/ijms22179554 (PMC8430575; doi:10.3390/ijms22179554)
Supplement: Supplementary file 1 [file ijms-22-09554-s001.zip › ijms-1336270-supplementary.pdf]

Supplementary Information:

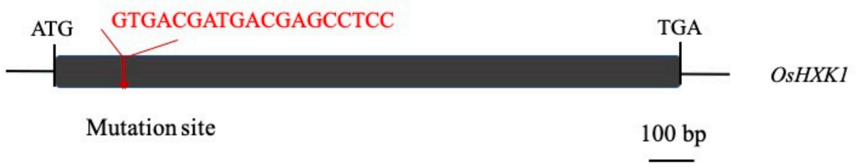

HHZ GTGACGATGACGAGCCTCCGGGAGGGCTGCGCTTGCGCGG
CH1/HZ GTGACGATGACGAC*CTCCGGGAGGGCTGCGCTTGCGCGG (substitution and deletion)
CH5/HZ GTGACGATGACGAGACCTCCGGGAGGGCTGCGCTTGCGCGG (insertion)
MXZ CGGCGGTGGCGGCAGATCAGAAGGTGGTGACGATGACGAGCCTCCGGGAGGGCTGCGCTTGCGCGG
CH1/MZ GCGGCGGTGGCGGCAGATCAGAAGGTGGTGTCGATGACGAACCTCCGGGAGGGCTGCGCTTGCGCGG (insertion and substitution)
CH3/MZ CGGCGGTGGCGGCAGATCAGAAGGTGGT ** CGATGACGAGCCCTCCGGGAGGGCTGCGCTTGCGCGG (deletion)
WSM GTGACGATGACGAGCCTCCGGGAGGGCTGCGCTTGCGCGG
CH2/W G**ACGATGACGAGCCTCCGGGAGGGCTGCGCTTGCGCGG (insertion and deletion)
CH3/W GTGACGATGACCTCGATGACG GAGCCTCCGGGAGGGCTGCGCTTGCGCGG (insertion)

Figure S1. Mutation analysis of *OsHXK1*-Cas9-transformed plants of the three varieties. * represents a deletion, and A, T, G, and C represent inserted and mutated bases, respectively.

**Target site**		GGTGACGATGACGAGCCTCC **GGG**			
**chromosome**	**position**	**sequence**	**off score**	**gene**	**No. of plants with mutation**
chr02	24754156	GATGACGAGGATGTGCCTCC**GGG**	0.15	Os02g0621800	0
chr12	22482824	GGTGACGATGAACAGCACCC**GGG**	0.06	Os12g0553050	0
chr12	19199542	GGTGACGCTGGAGAGGCTCC**GGG**	0.031	Os12g0503200	0
chr06	8876530	GGGGAAGATGACGCTCCTCC**GGG**	0.014	Os06g0267400	0
chr04	25341229	GGCGACGATGACGAGCCGCG**CGG**	0.008	Os04g0507100	0
chr04	28854194	GACGACGAGGACGAGCCTCC**GGC**	0.005	Os04g0572800	0
chr03	35429871	GGTGTCGCCGACGAGCCTCC**GGC**	0.003	Os03g0842900	0
chr04	34596920	GGCGTCGATGGCGAGCCTCC**GGT**	0.002	Os04g0677200	0

Figure S2. Analysis of missed detection of *OsHXK1* targets in *OsHXK1*-Cas9-transformed plants. The green bases represent protospacer adjacent motif (PAM) sites.

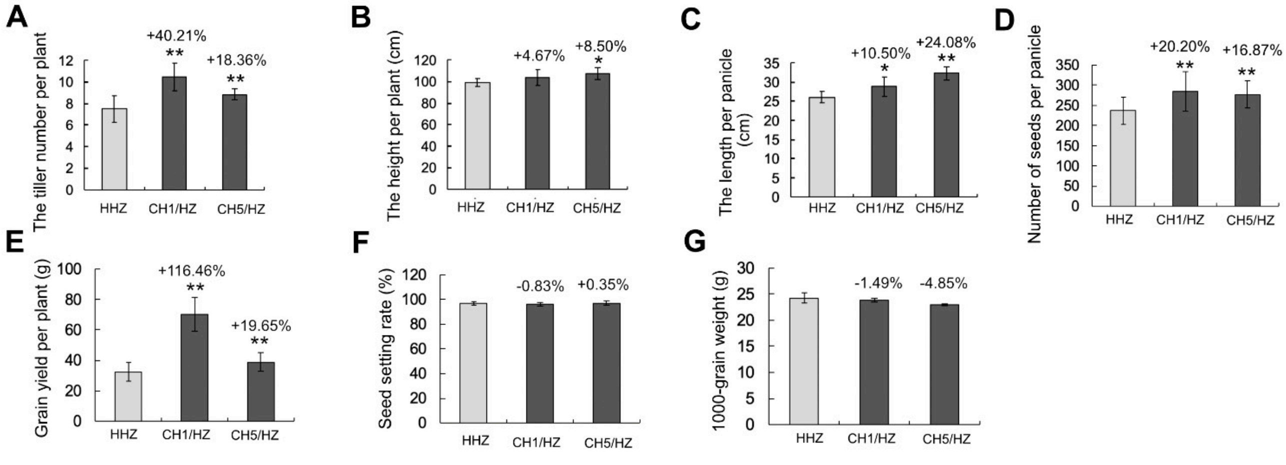

Figure S3. Yield traits of HHZ and *OsHXK1-Cas9* plants seeded in the spring.

(**A**) Tiller number of HHZ, CH1/HZ, and CH5/HZ plants. (**B**) Height of HHZ, CH1/HZ, and CH5/HZ plants. (**C**) Length per panicle of HHZ, CH1/HZ, and CH5/HZ plants. (**D**) Number of grains per panicle of HHZ, CH1/HZ, and CH5/HZ plants. (**E**) Single-plant yield of HHZ, CH1/HZ, and CH5/HZ plants. (**F**) Seed setting rate of HHZ, CH1/HZ, and CH5/HZ plants. (**G**) 1000-grain weight of HHZ, CH1/HZ, and CH5/HZ plants. Shown are the means ± SDs; *, $0.01 \leq P < 0.05$; **, $0.001 \leq P < 0.01$, according to Student's *t*-test.

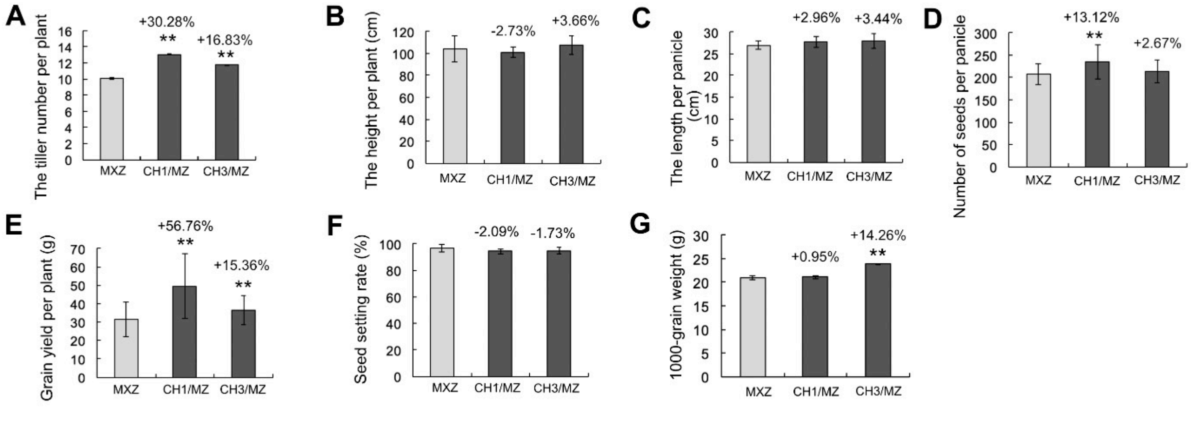

Figure S4. Yield traits of MXZ and *OsHXK1-Cas9* plants seeded in the spring.

(**A**) Tiller number of MXZ, CH1/MZ, and CH3/MZ plants. (**B**) Height of MXZ, CH1/MZ, and CH3/MZ plants. (**C**) Length per panicle of MXZ, CH1/MZ, and CH3/MZ plants. (**D**) Number of grains per panicle of MXZ, CH1/MZ, and CH3/MZ plants. (**E**) Single-plant yield of MXZ, CH1/MZ, and CH3/MZ plants. (**F**) Seed setting rate of MXZ, CH1/MZ, and CH3/MZ plants. (**G**) 1000-grain weight of MXZ, CH1/MZ, and CH3/MZ plants. Shown are the means ± SDs; *, $0.01 \leq P < 0.05$; **, $0.001 \leq P < 0.01$, according to Student's *t*-test.

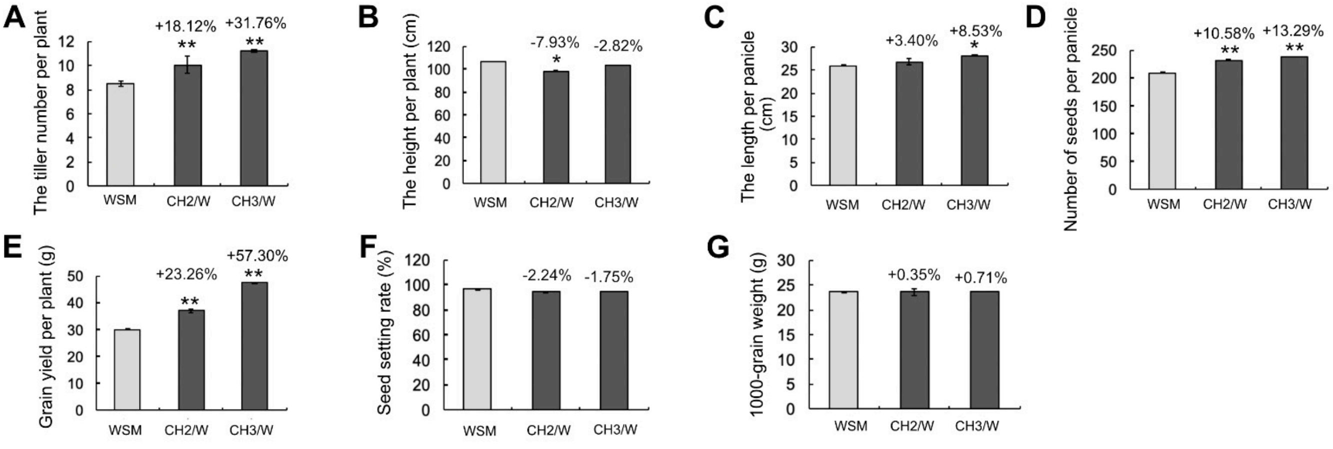

Figure S5. Yield traits of WSM and *OsHXK1-Cas9* plants seeded in the spring.

(**A**) Tiller number of WSM, CH2/W, and CH3/W plants. (**B**) Height of WSM, CH2/W, and CH3/W plants. (**C**) Length per panicle of WSM, CH2/W, and CH3/W plants. (**D**) Number of grains per panicle of WSM, CH2/W, and CH3/W plants. (**E**) Single-plant yield of WSM, CH2/W, and CH3/W plants. (F) Seed setting rate of WSM, CH2/W, and CH3/W plants. (**G**) 1000-grain weight of WSM, CH2/W, and CH3/W plants. Shown are the means ± SDs; *, $0.01 \leq P < 0.05$; **, $0.001 \leq P < 0.01$, according to Student's *t*-test.

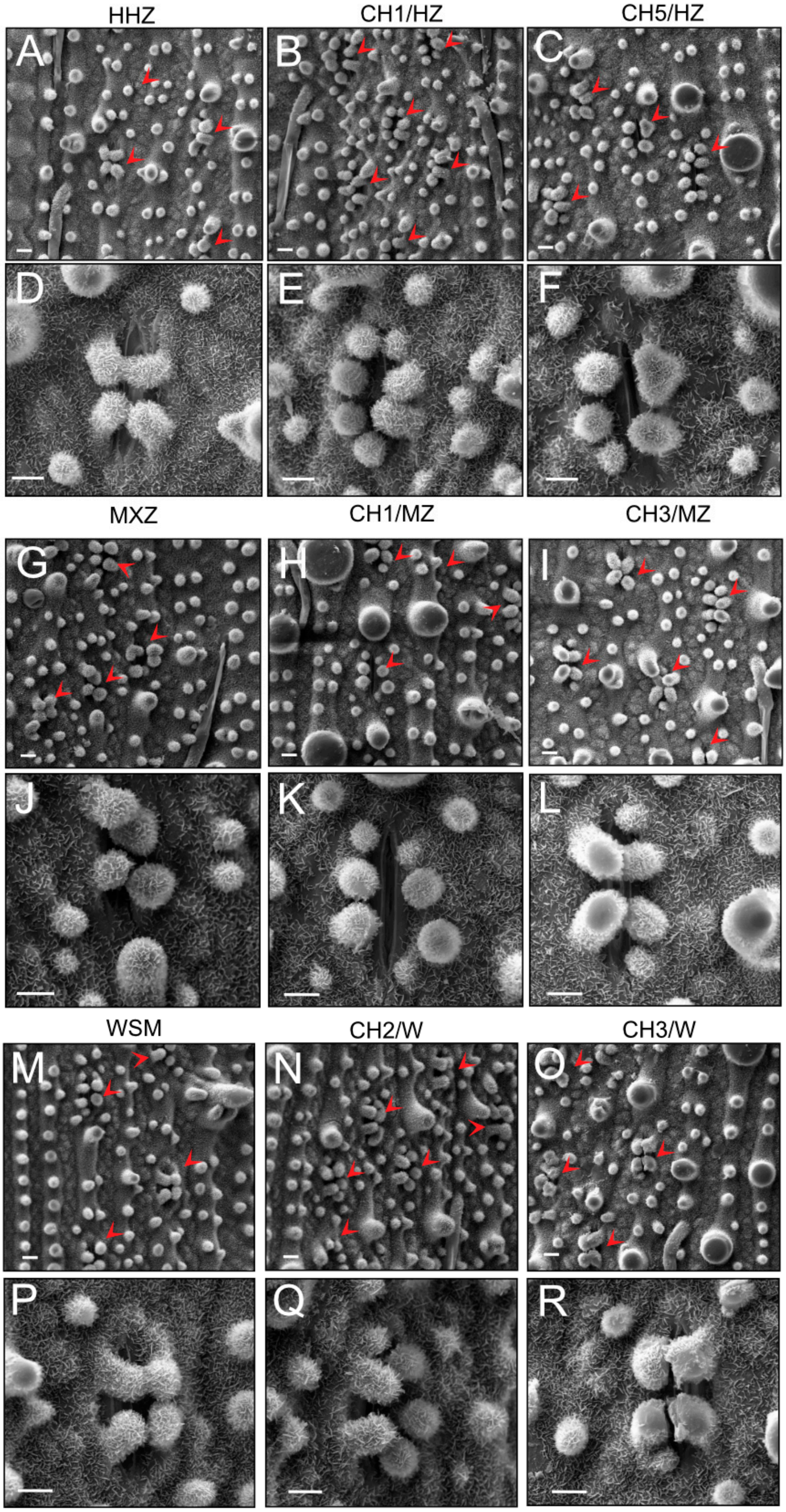
HHZ
CH1/HZ
CH5/HZ
A
B
C
D
E
F
MXZ
CH1/MZ
CH3/MZ
G
H
I
J
K
L
WSM
CH2/W
CH3/W
M
N
O
P
Q
R

Figure S6. The scanning electron microscopy (SEM) images of stomatal apertures in *OsHXK1-Cas9* plants. (**A**) – (**C**), (**G**) – (**I**), (**M**) – (**O**), The SEM observation (3000×) on stomata of outer epidermis under leaves of wildtype and the *OsHXK1-Cas9* plants. The stomata are marked by the red triangle. Bars = 2 μm. (**D**) – (**F**), (**J**) – (**L**), (**P**) – (**R**), The SEM observation (10,000×) observation on stomata of outer epidermis under leaves of wildtype and the *OsHXK1-Cas9* plants. Bars = 2 μm.

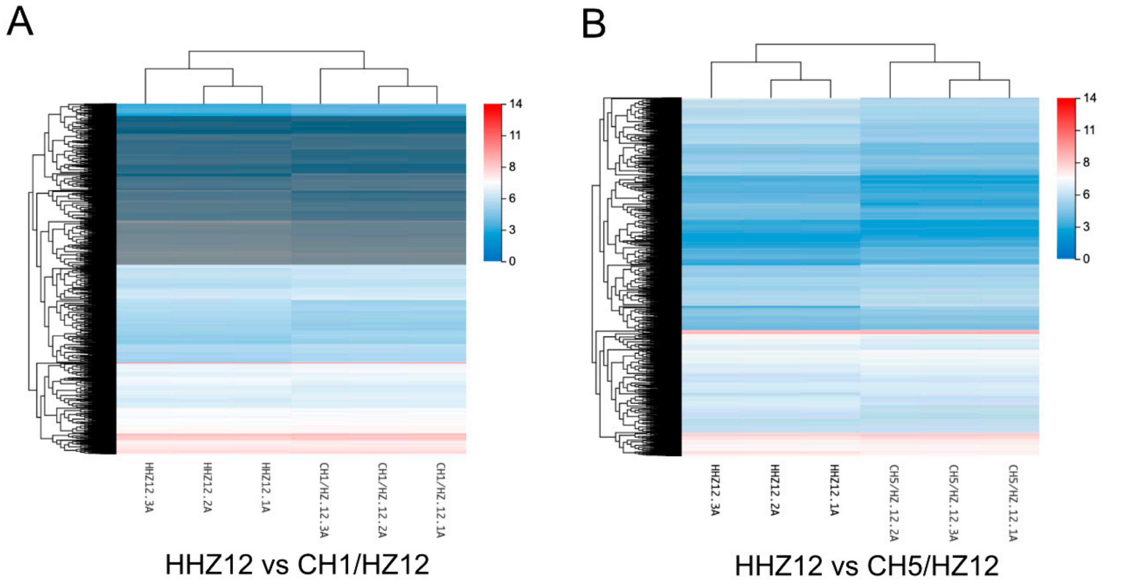

Figure S7. Cluster heat maps of DEGs in HHZ12-CH1/HZ and HHZ12-CH5/HZ

(**A**) Cluster heat maps of DEGs in the HHZ12 vs CH1/HZ comparison group. (**B**) Cluster heat map of DEGs in the HHZ12 vs CH5/HZ comparison group. The horizontal axis represents the log_2(FC) (expression value + 1), and the vertical axis shows the genes. Under the default color matching, the redder the color is, the higher the expression, and the bluer the color is, the lower the expression.

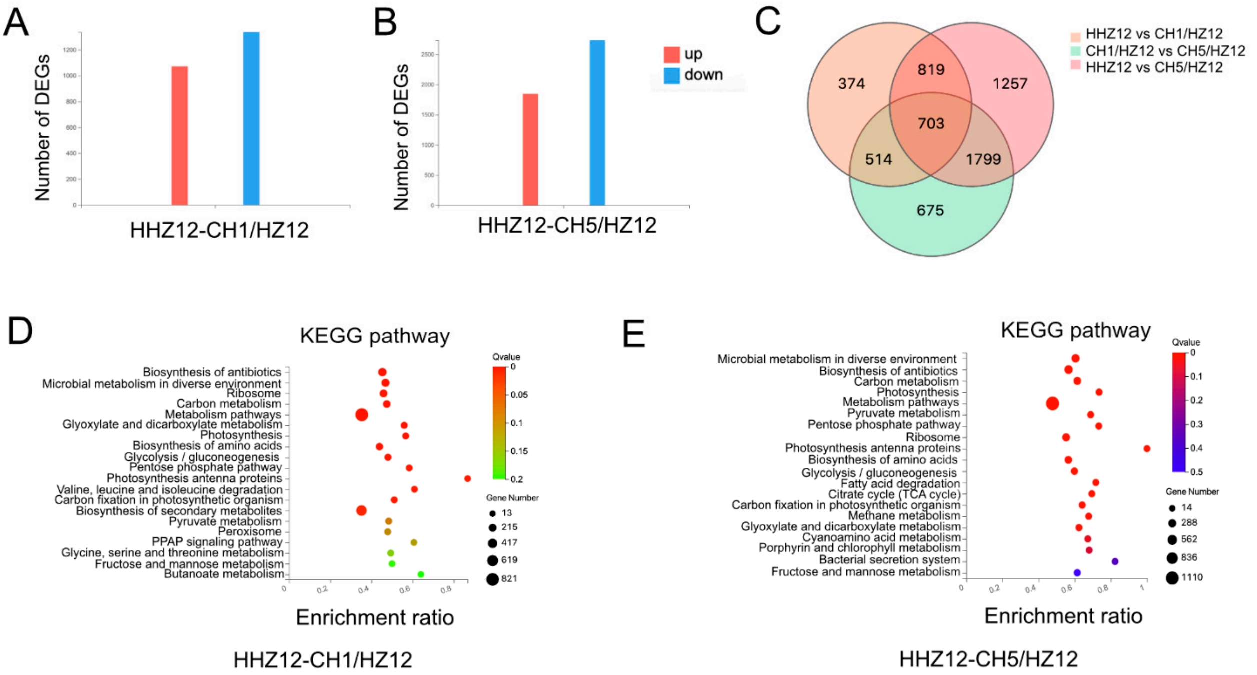

Figure S8. Analysis of DEGs screened by transcriptome sequencing. (**A**)–(**B**) The number of up- and downregulated DEGs ($log_2(FC) \geq 0.6$) in the HHZ12-CH1/HZ and HHZ12-CH5/HZ comparison groups. (**C**) Venn diagram showing the number of common DEGs in the HHZ12-CH1/HZ and HHZ12-CH5/HZ comparison groups ($log_2(FC) \geq 0.6$). (**D**)–(**E**) KEGG analysis of DEGs in the HHZ12-CH1/HZ and HHZ12-CH5/HZ comparison groups. The X-axis represents the enrichment ratio (the ratio of the number of genes annotated to an entry in the selected gene set to the total number of genes annotated to the entry in the species; the formula is Enrichment ratio = Term candidate gene number/Term gene number), the Y-axis lists the KEGG pathways, the size of the bubbles represents the number of genes annotated to each KEGG pathway, and the color represents the enrichment significance value (Q value). The redder the color is, the smaller the significance value.

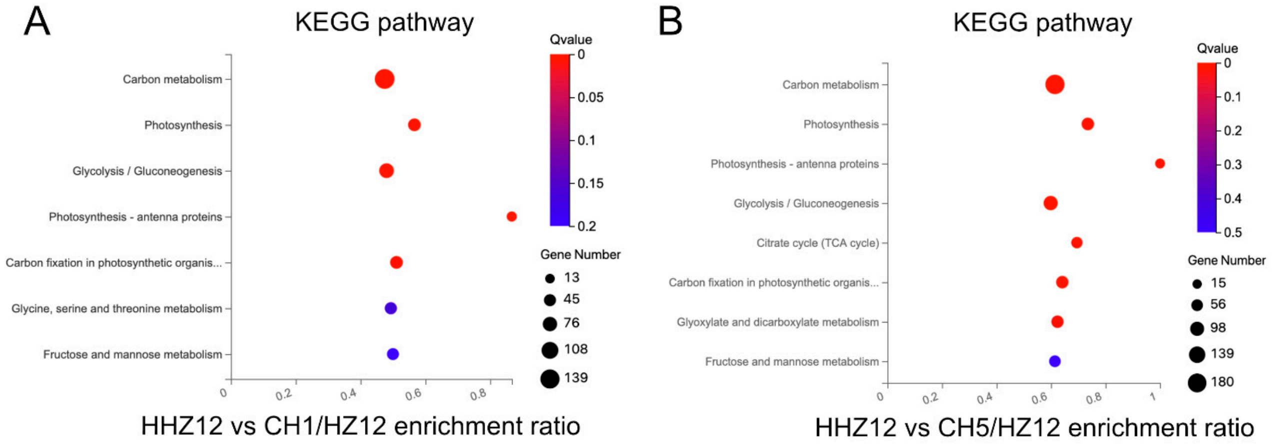

Figure S9. Kyoto Encyclopedia of Genes and Genomes (KEGG) pathway analysis of DEGs associated with photosynthesis-related metabolic pathways in the HHZ12 vs CH1/HZ and CH5/HZ comparison groups. (**A**)–(**B**) KEGG pathway analysis of DEGs in the HHZ12 vs CH1/HZ and CH5/HZ comparison groups. The X-axis represents the enrichment ratio (the ratio of the number of genes annotated to an entry in the selected gene set to the total number of genes annotated to the entry in this species; the formula is Enrichment ratio = Term candidate gene number/Term gene number), the Y-axis lists the KEGG pathways, the size of the bubbles represents the number of genes annotated to each KEGG pathway, and the color represents the enrichment significance value (Q value). The redder the color is, the smaller the significance value.

A

Group	29	54	102	263	316
I	D	M	F	S	G
II	A	T	F	A	G
III	A	T	Y	A	G
IV	A	T	F	S	R

B

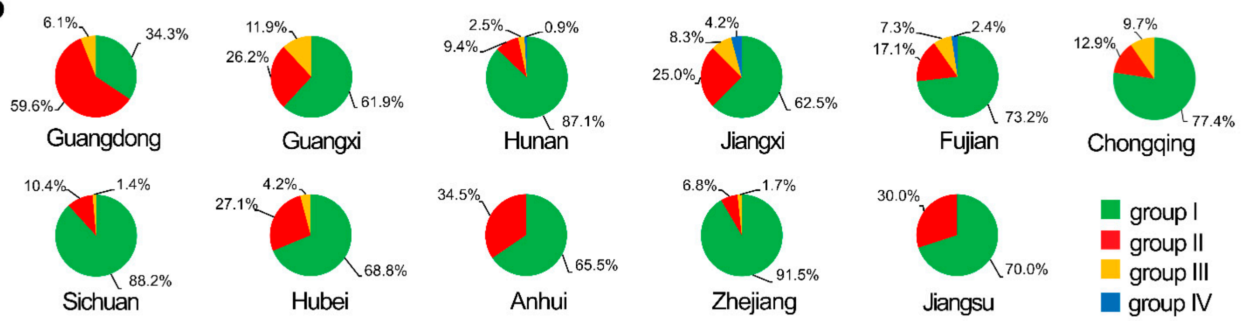

Figure S10. Haplotype distribution percentage of *OsHXK1* in eleven provinces of China. (**A**) The distribution type groups of *OsHXK1* haplotype analysis. (**B**) The Haplotype distribution percentage of *OsHXK1* in eleven provinces of China.

Table S1. *OsHXK1-Cas9* HHZ rice plant quality analysis.

lines	Head rice recovery (mm)	Amylose (%)	Milled rice rate (%)	Brown rice rate (%)	Protein (%)	Clarity
HHZ	70.00 ± 4.00	18.90 ± 0.30	67.00 ± 0.50	78.10 ± 0.60	7.76 ± 0.10	/
CH1/HZ	73.00 ± 3.00	18.90 ± 0.10	65.00 ± 0.20	79.20 ± 0.50	8.40 ± 0.10	Level 1
CH5/HZ	60.00 ± 2.00	18.60 ± 0.10	66.30 ± 1.00	78.50 ± 0.40	9.11 ± 0.10	Level 1

Table S2. MXZ *OsHXK1-Cas9* rice plant quality analysis.

lines	Head rice recovery (mm)	Amylose (%)	Milled rice rate (%)	Brown rice rate (%)	Protein (%)	Clarity
MXZ	67.00 ± 6.00	17.80 ± 0.20	65.30 ± 1.10	78.40 ± 0.30	8.20 ± 0.20	Level 1
CH1/MZ	64.00 ± 4.00	18.90 ± 0.10	66.00 ± 1.40	78.40 ± 0.40	8.35 ± 0.10	Level 1
CH3/MZ	74.00 ± 0.00	17.20 ± 0.40	65.50 ± 1.20	78.80 ± 0.60	7.89 ± 0.40	Level 1

Table S3. WSM *OsHXK1-Cas9* rice plant quality analysis.

lines	Head rice recovery (mm)	Amylose (%)	Milled rice rate (%)	Brown rice rate (%)	Protein (%)	Clarity
WSM	55.00 ± 3.00	17.50 ± 0.10	66.30 ± 0.50	79.70 ± 0.50	9.00 ± 0.10	/
CH2/W	56.00 ± 1.00	17.40 ± 0.30	68.00 ± 1.10	78.60 ± 0.40	9.31 ± 0.20	Level 1
CH3/W	59.00 ± 1.00	17.90 ± 0.40	65.50 ± 0.70	79.40 ± 0.40	9.10 ± 0.30	Level 1

Table S4. Photosynthesis-related genes in the CH1/HZ and CH5/HZ comparison groups.

**Gene ID**	**$\log_2$(CZ1-12/HHZ12)**	**$\log_2$ (CZ5-12/HHZ12)**	**Gene annotation**
4352014	0.74542	1.63064	Calvin cycle
4332690	0.77394	1.11244	Porphyrin and chlorophyll metabolism
4343515	0.6849	1.89106	Photosynthesis
4327544	0.61604	1.11339	Photosystem II oxygen-evolving complex
4332745	0.77453	1.47579	Photosystem II repair protein PSB27-H1

9271010	6.99173	7.90648	Phosphoglycolate phosphatase activity
4328925	1.07088	1.40265	Chloroplast thylakoid membrane protein complex
4327018	0.64306	0.69205	Chloroplast thylakoid membrane
9269069	1.08966	1.79622	Chloroplast outer membrane
4352264	0.61112	0.86897	NAD(P)H-quinone oxidoreductase, subunit U, chloroplastic
4347691	0.74734	0.93246	Triosephosphate isomerase, chloroplastic
4343189	0.70055	1.05024	Photosynthetic electron transport in photosystem I
4352021	1.15317	1.69537	Ribulose bisphosphate carboxylase
4331495	0.60145	0.9113	Glyceraldehyde 3-phosphate dehydrogenase, NAD(P)-binding domain
4326312	0.89629	1.63641	Glyoxylate reductase (NADP) activity
4347332	0.63786	0.95419	Transporter activity
112937008	1.02813	0.86603	RuBisCO large subunit
4351966	1.12168	1.37606	Ribulose bisphosphate carboxylase
Gene ID	$\log_2$(CZ1-12/HHZ12)	$\log_2$(CZ5-12/HHZ12)	Gene annotation
4330512	0.84151	1.74334	Pyrophosphate-fructose 6-phosphate 1-phosphotransferase subunit alpha
4333006	0.81252	0.70941	Metal ion transport
4349044	0.87747	1.10369	Glutamyl-tRNA reductase, chloroplastic-like
4335799	0.62521	1.33612	Photosystem I PsaO
4324705	2.26762	2.56028	Chlorophyll a-b binding protein of LHCII type I
4336044	0.7	0.74725	Glyceraldehyde 3-phosphate dehydrogenase
4349114	0.81938	0.9519	Glycine cleavage system H-protein
4336886	0.7206	1.01268	Thylakoid lumenal 29 kDa protein, chloroplastic
4343721	0.6138	0.68718	Geranyl diphosphate biosynthetic process
4336028	0.65434	1.20113	Photosynthesis, light harvesting
4328586	0.8363	1.26754	Cell redox homeostasis
4329999	1.17019	1.67652	Electron transfer activity
4326594	0.7065	0.90261	Glucose-1-phosphate adenylyltransferase activity
4344439	0.86115	1.05125	Ferredoxin-1, chloroplastic-like
4342001	0.74165	1.2575	Photosystem II repair
4330835	1.49552	2.2451	Ferredoxin-nitrite reductase

$\log_2$(FC) ≥ 0.6 was used as the screening standard for common DEGs.

Table S5. Haplotype distribution of *OsHXK1* rice varieties in China.

Province	Group I	Group II	Group III	Group IV	Sum
Guangdong	34	59	6	0	99
Guangxi	26	11	5	0	42
Fujian	60	14	6	2	82
Jiangxi	30	12	4	2	48

Hunan	277	30	8	3	318
Zhejiang	54	4	1	0	59
Anhui	19	10	0	0	29
Hubei	33	13	2	0	48
Jiangsu	21	9	0	0	30
Chongqing	24	4	3	0	31
Sichuan	127	15	2	0	144
Total	705	181	37	7	930

Table S6. Primers used in this study.

Primer name	Sequence (5'-3')	Objective
HXH1cas9-U3F	GTTGGTGACGATGACGAGCCTCC	Vector construction
HXH1cas9-U3R	AAACGGAGGCTCGTCATCGTCAC	Vector construction
Actin1F	CACATTCCAGCAGATGTGGA	qRT-PCR
Actin1R	GCGATAACAGCTCCTCTTGG	qRT-PCR
UQ5F	ACCTTCATGGCCAACCACTT	qRT-PCR
UQ5R	CTAAGCCTGCTGGTTGTAGA	qRT-PCR
GAPDHF	TACGTTGAGGAGGACCTTG	qRT-PCR
GAPDHR	GTCATACCAAGACACAAGCT	qRT-PCR
RbcS3F	ACCATCTCAATGGCCTCTGC	qRT-PCR
RbcS3R	TGTGTGCATATAGCCGGAGC	qRT-PCR
LOC_Os07g36080F	ACCAAATCTGCTCTTGGCGA	qRT-PCR
LOC_Os07g36080R	ACATGGAGGGTACATTGCCG	qRT-PCR
psbPF	AACGCCACGATTGAGGACAT	qRT-PCR
psbPR	TCGATCTTCTCCACGGAGGT	qRT-PCR
LOC_Os02g15750F	AGAGCCAACCAGCAATCACA	qRT-PCR
LOC_Os02g15750R	GCTGCCTCTGTACTGGTCTC	qRT-PCR
LOC_Os12g31460F	CCAACAAAGCTGGTTGGCTAC	qRT-PCR
LOC_Os12g31460R	CAGAGCATTCATGGTGACGC	qRT-PCR
LOC_Os07g28610F	CCATCGACAGGCTTCCACTT	qRT-PCR
LOC_Os07g28610R	CACCAGAGCTTCCCTGTCTG	qRT-PCR

OsRBCS4F	AACGTTAGGCAGGTGCAGTT	qRT-PCR
OsRBCS4R	TGCAGCTTAACACGGACACA	qRT-PCR
OsRBCS2F	AGTCTGGTGGCAACTAAGCC	qRT-PCR
OsRBCS2R	GCACGGCCGGTAAAATCAAA	qRT-PCR
LOC_Os04g33830 F	TTCTGGCTGTGGTTGGTGAC	qRT-PCR
LOC_Os04g33830 R	ATCCGTCCGTACAGTCAAGC	qRT-PCR
CP24-F	CCTTAACCTTAACGGCCATTTCC	qRT-PCR
CP24-R	CCCGCAGTAAAATCCTGAGC	qRT-PCR
OsFd1F	AGCAACAAGCTGGGAGACAG	qRT-PCR
OsFd1R	GAGTAAGGCAGGTCGATCCC	qRT-PCR
OsRBCS5F	GGAGTCCGGCGGAAACTAAG	qRT-PCR
OsRBCS5R	GGAAACCAATGCAAGGTGGC	qRT-PCR
casHXK1-SF	CGATGACGAGCCTCCGGGAG	Sequence
casHXK1-SR	GAACCCAAGCTCCCTCTCGC	Sequence
Os02g0621800F	CGCGCTGGCGCCCTCGCTCC	Sequence
Os02g0621800R	AGGCAGAAGACCTTGACGAC	Sequence
Os12g0553050F	ATGTTCTTGGATTCCAGGATC	Sequence
Os12g0553050R	TTAGATCCATGCCATCACAA	Sequence
Os12g0503200F	TATGAGCAGTCGCCCCATCG	Sequence
Os12g0503200R	ACCTTCTCCTCTTTCCTCCT	Sequence
Os06g0267400F	AGGACATGTTCGCGGCAGGC	Sequence
Os06g0267400R	TGAACTCCCCCGCGTCCTCCCA	Sequence
Os04g0507100F	GTCTCCGTCTTCGCGTTCCTC	Sequence
Os04g0507100R	GCCTCCGCTTCATCTTCCCAG	Sequence
Os04g0572800F	ATCCCTCCCTTAAACCAAGC	Sequence
Os04g0572800R	AGCGCGCACAGCAGCACGA	Sequence
Os03g0842900F	TCGCGTACGAGTACGCGCGGA	Sequence
Os03g0842900R	CATGAGGTGAGGGAACGCGA	Sequence
Os04g0677200F	AATGCCATCGGCGGCCAGAT	Sequence
Os04g0677200R	GCGGCGGGGAGCAGGTTGAA	Sequence
